# The Association between the Macronutrient Content of Maternal Diet and the Adequacy of Micronutrients during Pregnancy in the Women and Their Children’s Health (WATCH) Study 

**DOI:** 10.3390/nu4121958

**Published:** 2012-12-06

**Authors:** Michelle Blumfield, Alexis Hure, Lesley MacDonald-Wicks, Roger Smith, Stephen Simpson, David Raubenheimer, Clare Collins

**Affiliations:** 1 School of Health Sciences, University of Newcastle, Callaghan, New South Wales 2308, Australia; E-Mails: Michelle.Blumfield@newcastle.edu.au (M.B.); Lesley.Wicks@newcastle.edu.au (L.M.-W.); 2 Mothers and Babies Research Centre, Hunter Medical Research Institute, John Hunter Hospital, Level 3, Endocrinology, Locked Bag 1, Hunter Region Mail Centre, New South Wales 2310, Australia; E-Mail: Roger.Smith@newcastle.edu.au; 3 School of Medicine and Public Health, Faculty of Health, University of Newcastle, Callaghan, New South Wales 2308, Australia; E-Mail: Alexis.Hure@newcastle.edu.au; 4 School of Biological Sciences, University of Sydney, Sydney, New South Wales 2006, Australia; E-Mail: Stephen.Simpson@sydney.edu.au; 5 Institute of Natural Sciences, Massey University, Albany 0632, New Zealand; E-Mail: D.Raubenheimer@massey.ac.nz

**Keywords:** maternal, pregnancy, dietary intake, nutrition, nutrient requirements, protein

## Abstract

Nutrition during pregnancy can induce alterations in offspring phenotype. Maternal ratio of protein to non-protein (P:NP) energy has been linked to variations in offspring body composition and adult risk of metabolic disease. This study describes the dietary patterns of pregnant women by tertiles of the P:NP ratio and compares diet to Australian recommendations. Data are from 179 Australian women enrolled in the Women and Their Children’s Health Study. Diet was assessed using a validated 74-item food frequency questionnaire. Food group servings and nutrient intakes were compared to the Australian Guide to Healthy Eating and Australian Nutrient Reference Values. Higher maternal P:NP tertile was positively associated with calcium (*P* = 0.003), zinc (*P* = 0.001) and servings of dairy (*P* = 0.001) and meat (*P* = 0.001) food groups, and inversely associated with the energy dense, nutrient poor non-core (*P* = 0.003) food group. Micronutrient intakes were optimized with intermediate protein (18%E–20%E), intermediate fat (28%E–30%E) and intermediate carbohydrate (50%E–54%E) intakes, as indicated in tertile two. Results suggest a moderate protein intake may support pregnant women to consume the largest variety of nutrients across all food groups.

## 1. Introduction

Maternal nutrition during pregnancy can induce permanent alterations in offspring phenotype [1] and subsequently influence the risk of non-communicable diseases, such as obesity in adult life [2]. Variations in maternal macronutrient proportions have been shown to independently affect offspring outcomes, such as growth and body composition [3,4,5,6,7], insulin sensitivity [8], appetite [9], nutrient metabolism [9] and overall energy homeostasis [8] in both pre- and post-natal environments [10]. Effects are predicted to continue into adulthood and may have long-term consequences for the risk of metabolic disease [11]. 

However, the nutrient intakes required to optimize the short-term or long-term health of the offspring is undefined. Recent evidence has indicated that the ratio of protein to non-protein (P:NP) energy in the diet may play an important role in the development of obesity and subsequent non-communicable diseases [12]. This is due to regulatory mechanisms prioritizing the maintenance of an adequate protein intake level at the partial expense of carbohydrate and fat intake [12]. This phenomenon is explained by the protein leverage hypothesis [12], which may lead to overconsumption of carbohydrate coupled with increased energy intake, potentially leading to an increase in adiposity and an increased risk of obesity and metabolic disorders.

During pregnancy, protein availability is a key determinant of fetal growth [13]. Amino acids regulate pancreatic β cell differentiation, replication and insulin secretion [14]. There is currently insufficient evidence to provide a prescription for dietary protein content in pregnant women. However, studies suggest both maternal low protein [4,15,16,17] and high protein [3,17,18,19] intakes have poor outcomes for fetal body composition and metabolic health, such as restricted growth [3,17], increased adiposity [18], elevated cholesterol [4,16], elevated triglyceride [4,16] and leptin concentrations [4], impaired glucose tolerance [4,16] and insulin resistance [4,15].

We have recently shown in human pregnancy that the maternal macronutrient profile is associated with fetal adiposity and fat distribution [7]. Specifically, fetal abdominal subcutaneous fat was inversely associated with %E protein in the maternal diet, irrespective of whether fat or carbohydrate were the dilutents of protein [7]. This raises questions as to what these women were eating during pregnancy to provide these varying intakes of macronutrients.

While it is evident that macronutrients play an important function in the development of offspring adiposity, micronutrients also influence the regulation of body fat [20] and the aetiology of other health outcomes (e.g., folate and neural tube defects) [21]. However, the dietary patterns that pregnant women adopt to achieve different proportions of dietary protein and whether these eating patterns provide the essential micronutrients required for pregnancy is unclear. Therefore, the aim of this study was to: (i) describe the dietary patterns of pregnant women by tertiles of the P:NP ratio, and (ii) appraise the nutritional adequacy of eating patterns compared to Australian food group and micronutrient recommendations in order to provide evidence that may guide the development of an optimal P:NP ratio during pregnancy.

## 2. Experimental Section

### 2.1. Sample

Details of the sample have been previously described [7,22,23]. Briefly, the Women and Their Children’s Health (WATCH) Study is a prospective, longitudinal cohort that was initiated in July 2006 in Newcastle, Australia [22,24]. Of the 179 women recruited, 156 women who reported usual diet during pregnancy in the WATCH Study were included in the analysis. Ethics approval for the study was obtained from the Hunter New England Human Research Ethics Committee.

Demographic and social characteristics for the WATCH Study cohort and the sub-sample of pregnant participants with dietary data have been reported elsewhere [7,23]. Compared to the Australian population, the WATCH sample used in this analysis contained a higher proportion of women with post-year 12 higher school certificate qualifications (72.9% *vs.* 52.5%), a relative advantage based on postcode using the Index of Relative Socio-economic Advantage and Disadvantage (IRSAD) scale ≥5 (70.4% *vs.* 50.0%), but a similar proportion of overweight/obesity (46.2% *vs.* 44.0%) and indigenous ethnicity (3.1% *vs.* 2.5%) [7]. 

### 2.2. Data Collection

Dietary data were collected between 18 and 24 weeks and again at 36 to 40 weeks gestation using a validated 74-item food frequency questionnaire (FFQ), the Dietary Questionnaire for Epidemiological Studies. The tool was previously validated against weighed-food records in young women [25]. The FFQ includes food and beverage data, but does not ask about vitamin and mineral supplement use. The dietary intake reference period was the previous three months. Therefore, dietary data collected between 18 to 24 weeks and 36 to 40 weeks gestation referred to a reference period of 6 to 24 weeks gestation (early pregnancy) and 24 to 40 weeks gestation (late pregnancy), respectively. Positive moderate to strong pairwise correlations have been previously reported in this sub-sample between all dietary variables in early and late pregnancy (0.46 < *r* < 0.78; *P* < 0.001) [7]. Therefore, dietary intake during pregnancy was determined by averaging the reported early and late pregnancy intake data.

Food servings per day were calculated using portion sizes described in the Australian Guide to Healthy Eating (AGHE) [26], or standard portions derived from NUTTAB 2006, a national food composition database of Australian foods [27]. 

Questions on maternal demographic and social data were modeled on those in the Women’s Health Australia survey [28].

### 2.3. Australian Food and Nutrient Recommendations

Australia’s national food selection guide, the AGHE [26], was designed to encourage daily consumption from each of the five core food groups of breads/cereals (grains), lean meat and substitutes (including eggs, nuts and legumes), vegetables, fruit and dairy, in proportions that are consistent with the Dietary Guidelines for Australians [29]. A non-core food group contains energy dense, nutrient poor foods that do not belong to the core groups and are recommended to be consumed in limited amounts due to their high energy density and/or minimal nutrient contribution. Recommended servings for each food group have been developed for pregnant women [26].

Specific daily nutrient intake targets to optimize health and/or avoid nutritional deficiency have been recommended in the National Health and Medical Research Council of Australia nutrient reference values (NRVs) [30]. The most appropriate NRVs for comparison with population group intakes are estimated average requirements (EAR) and adequate intake (AI) [30]. The EAR is the daily nutrient level estimated to meet the requirements of half the healthy individuals in a particular life stage and gender group, with the proportion below the EAR providing a suitable approximation of the prevalence of inadequacy [30]. When an EAR is not able to be set, an AI is used instead, this being the average daily nutrient intake level that is assumed to be adequate [30].

### 2.4. Statistical Analysis

To improve the validity of the dietary analyses, the energy cut-off values recommended by Meltzer *et al.* (2008) were applied by excluding those who reported daily energy intakes <4.5 or >20.0 MJ/day (*n* = 7) [31]. Participants that remained (*n* = 149) were considered to have plausible dietary data.

Participants with plausible dietary data were divided into tertiles based on their reported P:NP ratio during pregnancy. The proportion of protein to carbohydrate and fat, each expressed as a percentage of total energy, was used to create P:NP tertiles.

Main outcome measures included maternal intake of vitamins, minerals and daily servings of food group intake. Dietary intake was also analyzed to determine those meeting AGHE and NRV dietary recommendations. Data were tested for normality. Normally distributed data are reported as mean (SD) and not normally distributed data as median (IQR). Multiple comparisons for the non-parametric dietary data were performed using the Kruskal-Wallis test. 

Parametric response surfaces for the median daily intake of each micronutrient were fitted over macronutrient intake arrays and then visualized by using nonparametric thin-plate splines [32]. This approach allowed the complex relationship between the response variable (each individual micronutrient) and the two major axes of % protein and % fat in the maternal diet to be visualized.

All data manipulation and statistical analyses were performed using Intercooled Stata 11.0 (Stata, College Station, TX, USA) [33]. Graphics were performed using *R *software [34]. *P*-values <0.05 were considered statistically significant.

## 3. Results

Maternal characteristics and pregnancy outcomes for the sub-group of women with plausible dietary data are presented in Table 1. Differences between women in the WATCH cohort with available dietary data (*n* = 156) and women without dietary data (*n* = 23) have been previously reported [7]. Briefly, women who reported dietary data were more likely to be married or in a *de facto *relationship (*P* = 0.03) and less likely to be at socio-economic disadvantage (*P* = 0.04) [7]. No significant differences were found between women with plausible (*n* = 149) and implausible dietary data (*n* = 7).

Table 2 reports the dietary composition during pregnancy for participants in the WATCH Study by tertile of the P:NP ratio. Pregnant women in the high P:NP ratio group achieved a higher P:NP ratio compared to women in the low P:NP ratio group by consuming a reduced total amount of carbohydrate and increased quantity of protein, as opposed to any changes in total fat intake (Table 2). Micronutrient intakes of calcium (*P* < 0.01) and zinc (*P* < 0.01) were positively associated with P:NP tertile, while for vitamin C (*P* = 0.008) and vitamin E (*P* = 0.003), there was a negative association with P:NP tertile (Table 2). 

Table 3 reports the daily food group servings of women during pregnancy, by tertile of the P:NP ratio. Pregnant women in high P:NP ratio group reported greater median daily servings of dairy (2.1 *vs.* 1.8; *P* < 0.001) and meat (2.0 *vs.* 1.4; *P* < 0.001) and lower servings of fruit (1.7 *vs.* 2.6; *P* = 0.014) and extras (3.6 *vs.* 4.6; *P* = 0.003) compared to women in the low P:NP ratio group. While significantly lower intakes of sweet non-core foods were reported by women in the high P:NP ratio group (1.2 *vs.* 1.5; *P* = 0.003), lower servings of savory non-core foods (2.1 *vs.* 2.6) also contributed to the decrease in extra servings. Pregnant women in medium P:NP ratio group reported the highest intake of fruit (2.7 servings) and vegetables (2.4 servings). 

**Table 1:** Maternal characteristics and pregnancy outcomes for the participants in the WATCH study with plausible dietary data, by tertile of the protein to non-protein ratio (*n* = 149) ^1,2^.

Characteristic	Low P:NP ratio	Medium P:NP ratio	High P:NP ratio	All women
	0.21 (0.19, 0.22) ^3^	0.24 (0.23, 0.25)	0.28 (0.27, 0.30)	0.24 (0.21, 0.27)
	*n* = 50	*n* = 50	*n* = 49	*n* = 149
Age (year)	27.8 ± 5.2 ^4^	29.6 ± 5.7	29.5 ± 5.5	29.0 ± 5.5
Height (cm)	165.0 ± 6.4	166.1 ± 6.3	163.3 ± 6.8	164.8 ± 5.6
Born in Australia [*n* (%)] ^5^	49 (98.0)	45 (90.0)	45 (91.8)	139 (93.3)
Aboriginal, not Torres Strait Islander [*n* (%)]	2 (4.0)	0 (0)	3 (6.1)	5 (3.4)
Married or in *de facto* relationship [*n* (%)]	41 (82.0)	45 (90.0)	43 (87.8)	129 (86.6)
Education [*n* (%)] ^6^	28 (56.0)	43 (86.0)	40 (81.6)	111 (74.5)
Socioeconomic status, IRSAD ^7^ decile ≤5 [*n* (%)] ^8^	15 (30.0)	12 (24.0)	17 (34.7)	57 (38.3)
Smoked during pregnancy [*n* (%)]	7 (14.0)	3 (6.0)	5 (10.2)	15 (10.2)
Prepregnancy weight (kg)	64.5 (57.0, 79.0)	67.3 (56.0, 78.5)	65.0 (60.0, 80.0)	69.7 ± 17.0
Weight gain during pregnancy (kg)	12.9 ± 6.2	14.2 ± 7.8	12.6 ± 6.0	13.2 ± 6.7
Nulliparous [*n* (%)]	15 (30.0)	25 (50.0)	23 (46.9)	63 (42.3)
Preterm delivery before 37 weeks of gestation	0 (0)	7 (14.0)	6 (12.2)	13 (8.7)
Infant birthweight (g)	3608 (3185, 3910)	3575 (3100, 3980)	3420 (3049, 3718)	3500 (3100, 3820)

P:NP: protein to non-protein; ^1^ Plausible data is defined as energy intakes ≥4.5 and ≤20.0 MJ/d; ^2^ The proportion of protein to carbohydrate and fat, each expressed as a percentage of total energy, was used to create P:NP tertiles;. ^3^ Median: 25th and 75th percentiles in parentheses (all such values); ^4^ Mean ± standard deviation (all such values); ^5^ Other countries include England (*n* = 4), Belgium (*n* = 1), Canada (*n* = 1), Malaysia (*n* = 1), New Zealand (*n* = 1), Papua New Guinea (*n* = 1) and the United States (*n* = 1); ^6^ Maternal education level ≥ Australian year 12 high school certificate. Pregnant women in the low P:NP ratio group reported lower education compared to women in the medium P:NP ratio group (Kruskal-Wallis *P = *0.005); ^7^ IRSAD, index of relative socioeconomic advantage and disadvantage; ^8^ Relative disadvantage and lack of advantage based on postcode (IRSAD decile ≤5).

**Table 2:** Dietary composition during pregnancy for participants in the WATCH Study with plausible dietary data, by tertile of the protein to non-protein ratio (*n* = 149) ^1,2^.

Dietary composition	Low P:NP ratio ^3^		Medium P:NP ratio		High P:NP ratio		*P* ^4^
	0.21 (0.19, 0.22) ^4^		0.24 (0.23, 0.25)		0.28 (0.27, 0.30)		
	*n* = 50		*n* = 50		*n* = 49		
	Median	IQR	Median	IQR	Median	IQR	
**Macronutrients**							
Energy (kJ)	7316.6	5994.7–9527.9	8158.6	6483.2–9624.9	7294.7	5943.9–8872.4	
Protein (%E)	16.8 ^a,b^	15.9–17.2	18.8 ^a,c^	18.2–19.4	21.4 ^b,c^	20.7–22.5	<0.001 ^a,b,c^
Total fat (%E)	37.8	35.1–40.5	37.7	35.8–40.7	37.7	35.3–40.4	
Saturated fat (%E)	16.3	14.1–18.9	16.2	13.9–17.5	15.8	13.7–18.7	
Monounsaturated fat (%E)	12.5	11.7–13.5	13.1	12.2–14.1	13.3	12.3–14.6	
Polyunsaturated fat (%E)	5.0	4.1–6.7	5.2	4.1–6.7	5.0	4.0–5.8	
Total carbohydrate (%E)	43.5 ^a,b^	41.2–46.0	41.2 ^a,c^	38.7–43.4	39.0 ^b,c^	36.1–41.3	<0.001 ^a,b^, <0.01 ^c^
Sugars (%E)	20.1 ^a^	18.1–23.4	19.6	17.7–21.2	18.2 ^a^	15.7–21.0	<0.01 ^a^
Starch (%E)	23.0 ^a^	20.8–25.0	21.5	20.4–23.3	20.3 ^a^	18.4–21.7	<0.001 ^a^
Fiber (%E)	3.7	3.2–4.5	4.3	3.7–4.9	4.0	3.4–4.4	
Cholesterol (mg)	223.0 ^a^	170.9–319.8	300.5	210.3–350.2	273.1 ^a^	224.0–372.2	<0.01 ^a^
**Vitamins**							
Vitamin A (RE μg)	793.1	600.6–1025.1	860.8	707.8–1119.7	741.0	585.2–971.9	
Retinol (μg)	415.1	315.0–541.1	451.6	348.3–520.2	378.7	295.9–501.7	
β-carotene (μg)	2004.2	1454.1–2980.2	2322.1	1663.3–3254.4	2198.0	1460.1–2642.4	
Thiamin (mg)	1.6	1.3–2.0	1.7	1.3–2.2	1.5	1.2–1.9	
Riboflavin (mg)	2.4	1.9–3.1	2.6	2.1–3.6	2.5	2.1–3.2	
Niacin Equivalents (mg)	33.9	26.5–44.2	41.8	32.4–48.0	37.9	32.2–49.4	
Niacin (mg)	19.6	15.0–26.2	23.1	18.1–28.0	21.0	16.7–27.4	
Folate (µg)	279.7	205.2–356.0	298.1	243.2–355.4	244.4	205.3–350.0	
Vitamin C (mg)	161.9	90.9–207.2	153.2 ^a^	116.5–190.3	105.0 ^a^	81.4–166.9	<0.01 ^a^
Vitamin E (mg)	6.5	4.6–8.3	6.7 ^a^	5.5–8.2	5.3 ^a^	4.3–7.2	<0.01 ^a^
**Minerals**							
Calcium (mg)	867.2 ^a,b^	715.9–1006.9	965.6 ^a^	811.5–1305.6	1067.8 ^b^	826.0–1279.6	<0.01 ^a,b^
Iron (mg)	11.9	8.9–15.2	12.9	10.1–16.3	11.7	9.6–15.4	
Magnesium (mg)	259.6	202.2–322.0	291.5	240.9–361.0	272.2	218.2–356.0	
Phosphorus (mg)	1299.5	1093.0–1626.9	1588.8	1277.0–1911.0	1548.5	1254.4–1905.2	
Potassium (mg)	2586.	2026.8–3200.1	3010.9	2612.6–3505.5	2848.8	2204.8–3491.2	
Sodium (mg)	2255.9	1805.1–2868.3	2566.9	2091.6–3184.1	2404.6	2072.6–2941.5	
Zinc (mg)	9.2 ^a,b^	7.6–12.7	12.2 ^a^	9.3–14.3	11.1 ^b^	9.9–14.8	<0.01 ^a,b^

P:NP: protein to non-protein; IQR: interquartile range; %E: percentage of total energy; RE: retinol equivalents; ^1^ Plausible data is defined as energy intakes ≥4.5 and ≤20.0 MJ/day; ^2^ The proportion of protein (%E) to carbohydrate (%E) and fat (%E), was used to create P:NP tertiles; ^3^ Median: 25th and 75th percentiles in parentheses (all such values); ^4^* P*-values were obtained using the Kruskal-Wallis test to compare all tertile groups. Differences between groups are denoted by each letter.

**Table 3:** Daily food group consumption during pregnancy for participants in the WATCH Study with plausible dietary data, by tertile of the protein to non-protein ratio (*n* = 149) ^1,2^.

Food group servings ^3^	Recommended food group servings	Low P:NP ratio		Medium P:NP ratio		High P:NP ratio		*P* ^5^
		0.21 (0.19, 0.22) ^4^		0.24 (0.23, 0.25)		0.28 (0.27, 0.30)		
		*n* = 50		*n* = 50		*n* = 49		
		Median	IQR	Median	IQR	Median	IQR	
Breads/cereals (servings/day)	4–6	2.7	2.0–3.2	2.6	2.2–3.4	2.5	1.9–2.9	
Fruit (servings/day)	4	2.6	1.4–3.3	2.7 ^a^	2.0–3.5	1.7 ^a^	1.0–2.8	<0.01 ^a^
Vegetables (servings/day)	5–6	1.8 ^a^	1.2–2.5	2.4 ^a^	1.8–3.0	2.3	1.9–2.7	<0.01 ^a^
Dairy (servings/day)	2	1.8 ^a,b^	1.3–2.2	2.0 ^a^	1.7–2.6	2.1 ^b^	1.7–2.8	<0.01 ^a,b^
Meat and alternatives (servings/day)	1.5	1.4 ^a^	0.9–1.8	1.6	1.4–2.0	2.0 ^a^	1.5–2.5	<0.001 ^a^
Extras (servings/day)	0–2.5	4.6 ^a^	3.6–5.8	4.3	3.1–5.6	3.6 ^a^	2.6–5.5	<0.01 ^a^
Sweet (servings/day)		1.5 ^a^	1.1–2.1	1.2	0.7–1.8	1.2 ^a^	0.6–2.0	<0.01 ^a^
Savory (servings/day)		2.6	2.0–3.4	2.6	1.9–3.6	2.1	1.8–2.9	
Alcohol (servings/day)		0.02	0.0–0.05	0.03	0.0–0.11	0.03	0.0–0.18	

P:NP: protein to non-protein; IQR: interquartile range; %E: percentage of total energy; FFQ: food frequency questionnaire; ^1^ Plausible data is defined as energy intakes ≥4.5 and ≤20.0MJ/d; ^2^ The proportion of protein (%E) to carbohydrate (%E) and fat (%E), was used to create P:NP tertiles; ^3^ Serve size (FFQ categories) (a) Breads & Cereals: bread 60 g, cereal 40 g, cooked porridge 230 g, muesli 65 g, cooked rice/pasta/noodles (including lasagna) 180 g, dry biscuits 40 g; (b) Fruit: fruit whole (including canned fruit) 150 g, fruit juice 125 mL; (c) Vegetables: vegetable whole (including potatoes cooked without fat) 75 g; avocado 30, lettuce/endive/salad greens 36 g, tomato sauce/paste 20 g; (d) Dairy: milk 250 mL, cheese 40 g, yogurt 200 g, flavored milk 250 mL; (e) Meat & Alternatives: beef/veal/chicken/lamb/pork 85 g, fish (steamed/grilled/baked/canned) 100 g, ham 100 g, baked beans/tofu/soy beans/soy bean curd/other beans (including chickpeas, lentils, *etc.*) 80 g, nuts 40 g, eggs 100 g; (f) Extras: sweet biscuit 35 g, cakes/sweet pies/tarts/other sweet pastries 40 g, meat pies/pasties/quiche/other savory pies 60 g, pizza 60 g, hamburger 60 g, chocolate 25 g, peanut butter 25 g, potato crisps/corn chips/Twisties^®^ 30 g, jam/marmalade/honey/syrups 45 g, Vegemite^®^/Marmite^®^/Promite^®^ 100 g, ice-cream 50 g, bacon 50 g, corned beef/luncheon meats/salami 110 g, sausages/frankfurters 55 g, fried fish 65 g, fat spread 20 g, sugar 40 g, fries 60 g, light beer 600 mL, heavy beer 400 mL, wine (including sparkling wines) 200 mL, spirits/liqueurs 60 mL, fortified wines/port/sherry 60 mL; ^4^ Median; 25th and 75th percentiles in parentheses (all such values); ^5^* P*-values were obtained using the Kruskal-Wallis test to compare all tertile groups. Differences between groups are denoted by each letter.

**Figure 1:**
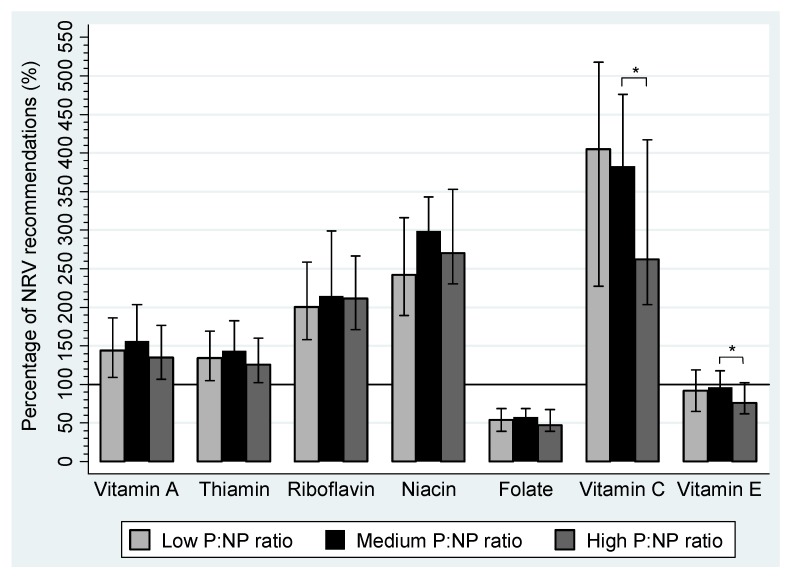
The percentage of Australian Nutrient Reference Value (NRV) recommendations for vitamins that were achieved during pregnancy by women in the WATCH Study who reported plausible dietary data (*n* = 149) by tertile of the protein to non-protein (P:NP) ratio. Plausible data is defined as energy intakes ≥4.5 and ≤20.0MJ/day. The proportion of protein (%E) to carbohydrate (%E) and fat (%E) was used to create P:NP tertiles. The P:NP ratio for each tertile: Low P:NP ratio group 0.21 (0.19, 0.22), *n* = 50; Medium P:NP ratio group 0.24 (0.23, 0.25), *n* = 50; high P:NP ratio group 0.28 (0.27, 0.30), *n* = 49. Nutrient Reference Values recommended for pregnancy: Vitamin A = 550 μg, Thiamine = 1.2 mg, Riboflavin = 1.2 mg, Niacin = 14 mg NE, Folate = 520 μg, Vitamin C = 40 mg, Vitamin E = 7 mg [30]. * *P* < 0.01.

**Figure 2:**
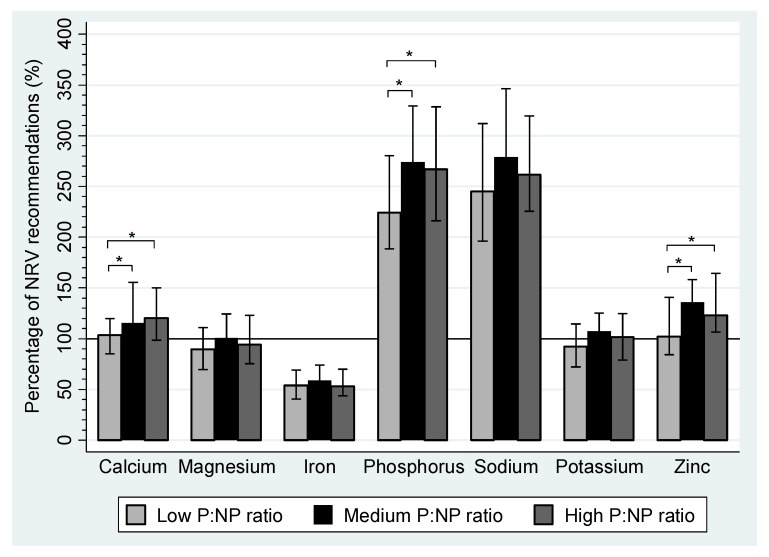
The percentage of Australian Nutrient Reference Value (NRV) recommendations for minerals that were achieved during pregnancy by women in the WATCH Study who reported plausible dietary data (*n* = 149) by tertile of the protein to non-protein (P:NP) ratio. Plausible data is defined as energy intakes ≥4.5 and ≤20.0 MJ/d. The proportion of protein (%E) to carbohydrate (%E) and fat (%E) was used to create P:NP tertiles. The P:NP ratio for each tertile: Low P:NP ratio group 0.21 (0.19, 0.22), *n* = 50; Medium P:NP ratio group 0.24 (0.23, 0.25), *n* = 50; high P:NP ratio group 0.28 (0.27, 0.30), *n = *49. Nutrient Reference Values recommended for pregnancy: Calcium = 840 mg, Magnesium = 290 mg, Iron = 22 mg, Phosphorus = 580 mg, Sodium = 920 mg, Potassium = 2800 mg, Zinc = 9 mg [30]. * *P* < 0.01; IQR: interquartile range.

The percentage of NRV recommendations for vitamins and minerals that were achieved by pregnant women are summarized in Figure 1 and Figure 2, respectively. Median daily intakes of vitamin A, thiamine, riboflavin, niacin, vitamin C, calcium, phosphorus, sodium and zinc were above 100% of the NRVs in each tertile. Magnesium and potassium intakes only achieved NRV recommendations in the medium P:NP ratio group and the medium and high P:NP ratio groups, respectively. Folate, vitamin E and iron intakes were suboptimal in all tertiles, with less than 60% of recommendations achieved for folate or iron and 75%–95% of NRVs met for vitamin E. Median daily intakes were the highest in the medium P:NP ratio group for all micronutrients, except vitamin C and calcium.

**Figure 3:**
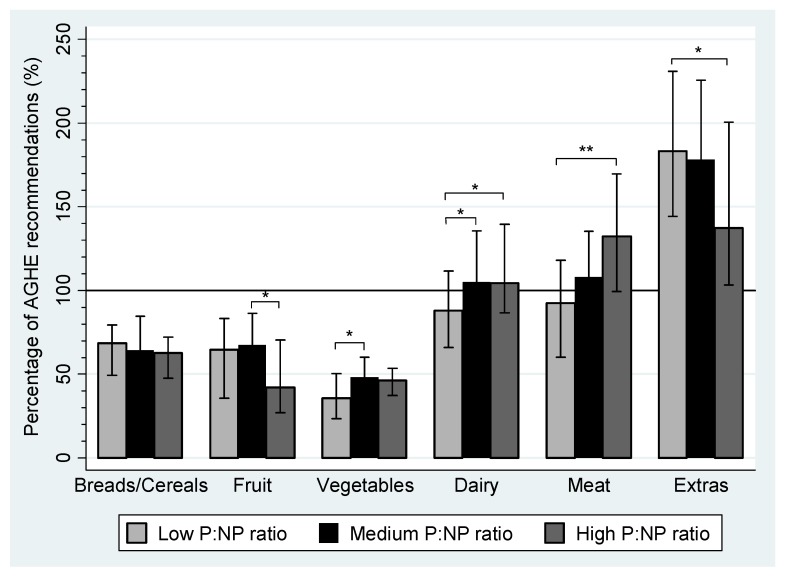
The percentage of Australian Guide to Healthy Eating (AGHE) food group recommendations achieved during pregnancy by women in the WATCH Study who reported plausible dietary data, by tertile of the protein to non-protein (P:NP) ratio. Plausible data is defined as energy intakes ≥4.5 and ≤20.0 MJ/day. The proportion of protein (%E) to carbohydrate (%E) and fat (%E) was used to create P:NP tertiles. The P:NP ratio for each tertile: Low P:NP ratio group 0.21 (0.19, 0.22), *n* = 50; Medium P:NP ratio group 0.24 (0.23, 0.25), *n* = 50; high P:NP ratio group 0.28 (0.27, 0.30), *n* = 49. AGHE minimum daily serving recommendations for pregnancy: Bread/Cereals = 4 servings, Fruit = 4 servings, Vegetables = 5 servings, Dairy = 2 servings and Meat = 1.5 servings. The maximum AGHE serving recommendation was used for the Extras food group = 2.5 servings [26]. * *P* < 0.01; ** *P* < 0.001; IQR: interquartile range.

Figure 3 summarizes the percentage of AGHE recommendations achieved by pregnant women according to tertile of the P:NP ratio. None of the women in any tertile achieved the AGHE recommendations for all food groups. The highest adherence rates were for dairy and meat food groups. Pregnant women in the medium and high P:NP ratio groups reported median daily servings of dairy and meat that were above minimum recommendations, while 88.1% and 92.4% of women in low P:NP ratio group met the recommendations, respectively. When food patterns in the low P:NP ratio group were compared to the high P:NP ratio group, adherence rates for dairy (88.1%* vs.* 104.5%; *P* = 0.001) and meat (92.4%* vs.* 132.2%; *P* < 0.001) food groups were lower, and for fruit (64.6%* vs.* 42.1; *P* = 0.014), higher for women in the low P:NP ratio group. Similar adherence rates were found for the bread and cereals food group. Servings of extra foods were compared to the maximum AGHE recommendation of 2.5 servings per day. Despite an inverse association between extra food servings and the P:NP ratio (*P* = 0.003), women in the high P:NP ratio group still reported intakes 37.3% above the maximum recommendation. 

**Figure 4:**
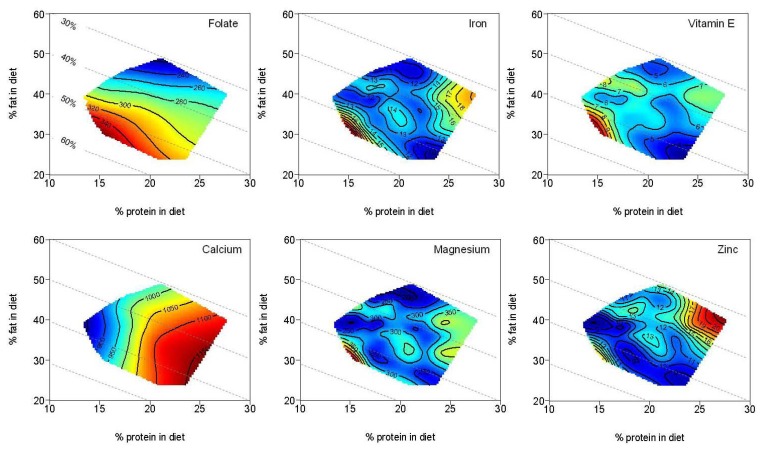
Effects of maternal macronutrient intakes during pregnancy on selected maternal micronutrient intakes. In these plots, known as right-angled mixture triangles [35], fat and protein (% of energy) increase along their respective axes in the normal way, and % carbohydrate decreases with distance from the origin. The negatively sloped dashed lines show the % carbohydrate, as labeled in the first graph. Plotted onto arrays of maternal dietary macronutrient composition points are fitted surfaces for the six response variables (folate, iron, vitamin E, calcium, magnesium and zinc). The isoclines for the micronutrient intakes rise in elevation from dark blue to dark red. Therefore, low micronutrient intakes are represented in the dark blue shading and increase to high micronutrient intakes in the red shading. Micronutrient intakes were generally optimized with a maternal diet of intermediate protein (18%E–20%E), intermediate fat (28%E–30%E) and intermediate carbohydrate (50%E–54%E) in comparison to Nutrient Reference Values (*n* = 149). Folate and iron intakes were maximized with low protein (<16%E), intermediate fat (30%E) and high carbohydrate (>54%E) intakes. Nutrient Reference Values recommended for pregnancy: Folate = 520 μg, Iron = 22 mg, Vitamin E = 7 mg, Calcium = 840 mg, Magnesium = 290 mg, Zinc = 9 mg [30].

Response surfaces for the effects of maternal macronutrient intake during pregnancy on selected micronutrient intakes (folate, iron, vitamin E, calcium, magnesium and zinc) are presented in Figure 4. Folate and iron intakes were maximized with low protein (<16%E), intermediate fat (30%E) and high carbohydrate (>54%E) intakes. Intakes of all remaining micronutrients (including those not shown) were optimized with a maternal diet of intermediate protein (18%E–20%E), intermediate fat (28%E–30%E) and intermediate carbohydrate (50%E–54%E). 

## 4. Discussion

This study provides the first insight into the eating patterns adopted by pregnant women by tertile of the P:NP ratio and compares patterns to Australian diet and nutrient recommendations. Pregnant women increased their P:NP ratio by an increased protein and reduced carbohydrate intake, as opposed to any changes in total fat intake. The P:NP ratio was positively associated with median daily servings of dairy and meat food groups and inversely associated with the extras food groups. While nutritional adequacy of micronutrient intakes did not significantly improve with increased P:NP tertile, women in the medium P:NP ratio group reported the highest median daily intake for all micronutrients, except vitamin C and calcium.

There was no evidence that a high P:NP ratio improved nutritional adequacy in pregnant women. The dietary patterns reported in this study were similar to those reported by a nationally representative sample of pregnant women in Australia [36]. Previous studies have reported a mismatch between the eating patterns of Australian women during pregnancy and food group recommendations [36], but suggest that an increased fruit and dairy intake may assist women to meet the NRVs for key nutrients important for childbearing (calcium, folate, iron, zinc and fiber) [36]. Results from the present study indicate that women in the medium P:NP ratio group (~18%E–19%E protein) consumed the highest median fruit intake, in conjunction with more vegetables, dairy and a trend towards more meat, compared to women in the low P:NP ratio group (~16%E–17%E protein); whereas women in the high P:NP ratio group (~21%E–22%E protein) consumed less fruit and extras, but more meat compared to those in medium P:NP ratio group. The reduced intake of energy dense, nutrient poor foods in the extras food group by women in the high P:NP ratio group may be beneficial for both maternal [37] and offspring body composition [7]. Plus, reduced intake of these extra foods may reduce offspring risk of obesity through the development of favorable alterations within the central neural network for appetite regulation [38]. However, a reduction of fruit intake without an equivalent increase in vegetable intake may be detrimental for micronutrient intakes, particularly folate. Fruits and vegetables contain many biologically active phytochemicals that have additive and synergistic effects that promote health and prevent chronic disease [39]. These properties are likely to have an important influence during critical windows of fetal development and may consequently affect offspring risk of disease later in life [1]. No significant differences in total fat, saturated fat or sugars were found between women in the medium and high P:NP ratio groups. Therefore, a moderate protein content, such as that adopted by women in medium P:NP ratio group (~18%E–19%E), may allow women to consume the largest variety of nutrients across all food groups. Further research to identify the optimal macronutrient composition during pregnancy and the implications on health in animal models and long-term epidemiological cohorts is required before dietary recommendations can be formulated for pregnant women.

The micronutrients at highest risk of deficiency during pregnancy in developed countries are folate and iron [40]. In this study, folate and iron intakes were well below recommendations in all tertiles of the P:NP ratio, despite higher intakes being visualized with lower protein contents. Results for folate were in agreement with those conducted in women where there was no relationship with diet composition [41]. But interestingly, iron intakes did not improve with increased P:NP ratio and the associated increased meat intake. This is likely to be because the decreased consumption of breads/cereals, fruit and extras servings reported with increased P:NP ratio reduced iron intakes by a similar magnitude. While some cross-sectional studies have reported a positive association between iron status and meat intake [42,43,44], recent data from a nationally representative sample in the United Kingdom have shown that iron fortified cereals were the largest contributors of iron intake in all age groups [45]. Iron fortified cereals were not specifically quantified by the FFQ used in this study. However, most Australian cereals now contain added iron, and this information was included in the nutrient composition database that was used to provide nutrient intakes in this study. Our results support these findings and indicate that women report inadequate intakes of folate and iron during pregnancy, regardless of whether they consume a protein-rich or carbohydrate-rich diet. While it is possible for pregnant women to achieve nutrient recommendations through food intake alone [36], pregnant women commonly report suboptimal nutrient intakes and may require dietary supplementation. No information was collected on vitamin supplementation in this study, because the purpose was to focus on nutrients supplied from food only. Thus, the nutritional adequacy of contemporary eating patterns across all food groups is as important as macronutrient balance in ensuring micronutrient intakes during pregnancy meet nutrient recommendations.

Our findings contribute to understanding the nutritional impact of maternal protein intake during pregnancy. The mechanisms connecting protein in maternal diet to obesity and metabolic disease in offspring are not fully elucidated, but are believed to involve changes in gene expression through epigenetic alterations, such as DNA methylation [46]. Research in experimental animal models has provided compelling evidence to support a role for glucocorticoids in this process [11,20]. Both maternal protein restriction and protein excess has been linked to increased glucocorticoid sensitivity in offspring, while increased glucocorticoid sensitivity in offspring has been associated with an increased risk of metabolic disease [11,20]. Experimental rodent models have also demonstrated that restriction of maternal micronutrients during pregnancy (multivitamin, multimineral and/or singular nutrients) can lead to an increase in offspring adiposity [20]. The specific phenotypic changes reported by rodent studies include an overall increase in percentage body fat, greater visceral/central fat accumulation, elevated expression of genes related to adiposity and modifications in adipose tissue function and lipid metabolism [20]. Results suggest there may be an optimal proportion of protein in maternal diet to maximize micronutrient adequacy during pregnancy. However, before an optimal macronutrient range can be recommended, research investigating the implications of these contemporary eating patterns on the offspring’s expected postpartum developmental environment is required.

Limitations include the use of self-reported FFQ data to measure dietary intake. Dietary data is strengthened by the similarities between the daily mean energy intake reported in our study (8070 kJ/day) and that reported in a representative sample of pregnant women in the Australian Longitudinal Study on Women’s Health (7795 kJ/day) in 2003 [47]. Macronutrient distributions were also similar to the Australian data [47]. No information was collected on vitamin supplementation, however, the main purpose was to focus on nutrients supplied from food only. Lastly, the WATCH study contained a higher proportion of women with post-school qualifications, socio-economic advantage and in a married or *de facto* relationship, but had a similar proportion of overweight/obesity and indigenous ethnicity compared to the Australian population. Therefore, care should be taken in extrapolating results at the population level.

## 5. Conclusions

This study provides evidence that there may be a target maternal macronutrient composition that optimizes micronutrient intakes during pregnancy. Despite eating patterns failing to meet food selection guide recommendations, micronutrient intakes for most nutrients were optimized at intermediate protein (18%E–20%E), intermediate fat (28%E–30%E) and intermediate carbohydrate (50%E–54%E) intakes. Further research is required to understand how contemporary eating behaviors can optimize micronutrient and macronutrient intakes during pregnancy to maximize the health of the mother and developing fetus. 
